# Right-sided descending colon with necrotizing enterocolitis: a rare case report

**DOI:** 10.3389/fped.2025.1557672

**Published:** 2025-06-06

**Authors:** Ting Wang, Shunlin Xia, Mengxu Liu, Youcheng Zhang, Yu Liu

**Affiliations:** Huai’an Maternal and Child Health Care Hospital Affiliated to Yangzhou University, Huai’an, China

**Keywords:** right-sided descending colon, necrotizing enterocolitis (NEC), newborn, end ileostomy, case report

## Abstract

**Background:**

Anatomical variations of the colon are commonly reported, with the majority involving the right colon. However, anomalies affecting the left colon, particularly the descending colon, are rarely described in the literature.

**Methods:**

We reviewed the clinical records of right-sided descending colon with necrotizing enterocolitis involvement at our hospital, detailing the patients’ onset, imaging studies, complications, and treatment.

**Results:**

A newborn experienced repeated vomiting. Upper and lower gastrointestinal imaging revealed gastroesophageal reflux, the right-sided descending and sigmoid colon. Two weeks later, the infant developed NEC, and after failure of conservative treatment, surgical intervention was performed. We found that the descending colon was fixed to the posterior abdominal wall, extending from the splenic flexure toward the right side and crossing the midline. The entire colon is dilated with thickened walls. Extensive mucosal ulceration is present, accompanied by transmural necrosis. Postoperative pathology reveals significant infiltration of inflammatory cells. The most severely affected regions were the descending and sigmoid colon. Consequently, a total colectomy with end ileostomy was performed, while the distal part of sigmoid colon was preserved. Postoperatively, the newborn recovered well.

**Conclusion:**

This case may help raise awareness among surgeons regarding the variability in the position of the descending colon. It is essential to consider such anatomical variations before performing related procedures in this area, to enhance surgical safety and avoid colonic injury.

## 1 Introduction

The normal descending colon is partially covered by and attached to the peritoneum. It begins at the left colic flexure, descends along the left lateral posterior wall of the abdominal cavity, and continues to the sigmoid colon at the level of the left iliac crest. However, in some individuals, variations in the position of the colon can occur, possibly due to differential development of abdominal organs and surrounding tissues or as a result of intestinal malrotation during embryonic development.

The sigmoid colon is the segment of the colon that exhibits the greatest variability in both length and position, especially in children, with a right-sided sigmoid colon occurring in approximately 35.16%–44% of cases (1, 2). Conversely, changes in the position of the descending colon are much rarer. A review of the relevant literature reveals that reports of a right-sided descending colon are mostly incidental (3, 4), and lesions involving the right-sided descending colon have not been documented. In this report, we describe the first case of a right-sided descending colon with necrotizing enterocolitis(NEC) in a newborn.

## 2 Case description

**Figure 1:**
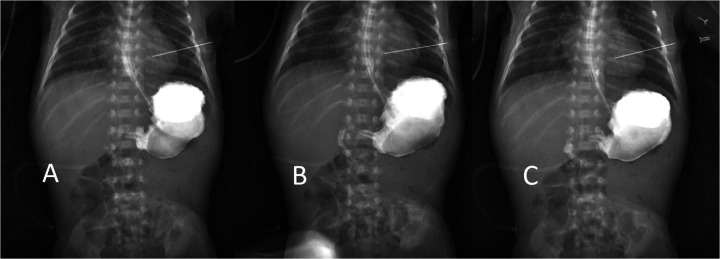
Lower gastrointestinal imaging shows that the frst and second parts of the duodenum are to the right of the midline, **(A,B)** and the duodenal—jejunal junction cross the midline **(C)**.

**Figure 2:**
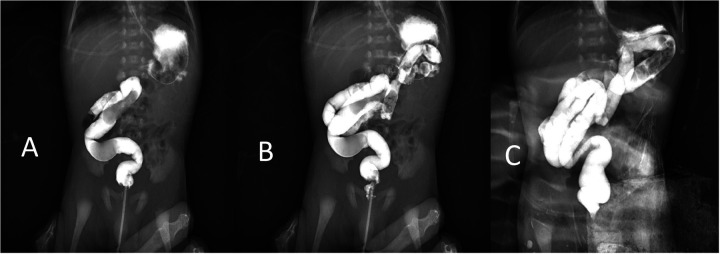
Lower gastrointestinal imaging showed the contrast agent into the sigmoid colon **(A)**, descending colon **(B)**, and transverse colon **(C)**. The descending colon moves to the right from the splenic flexure to the midline.

The male newborn was delivered by cesarean section at 35 weeks of gestation, with a birth weight of 2,250 g. Postnatally, the newborn experienced repeated vomiting, primarily consisting of semi-digested milk, with no bile observed. Ultrasound examination of the pylorus revealed no obvious abnormalities. Upper and lower gastrointestinal imaging showed signs of gastroesophageal reflux, right-sided descending and sigmoid colon (Figure 1,2). The newborn was positioned laterally with the upper body elevated during feeding. The vomiting subsided, and the volume of milk intake gradually increased.

**Figure 3:**
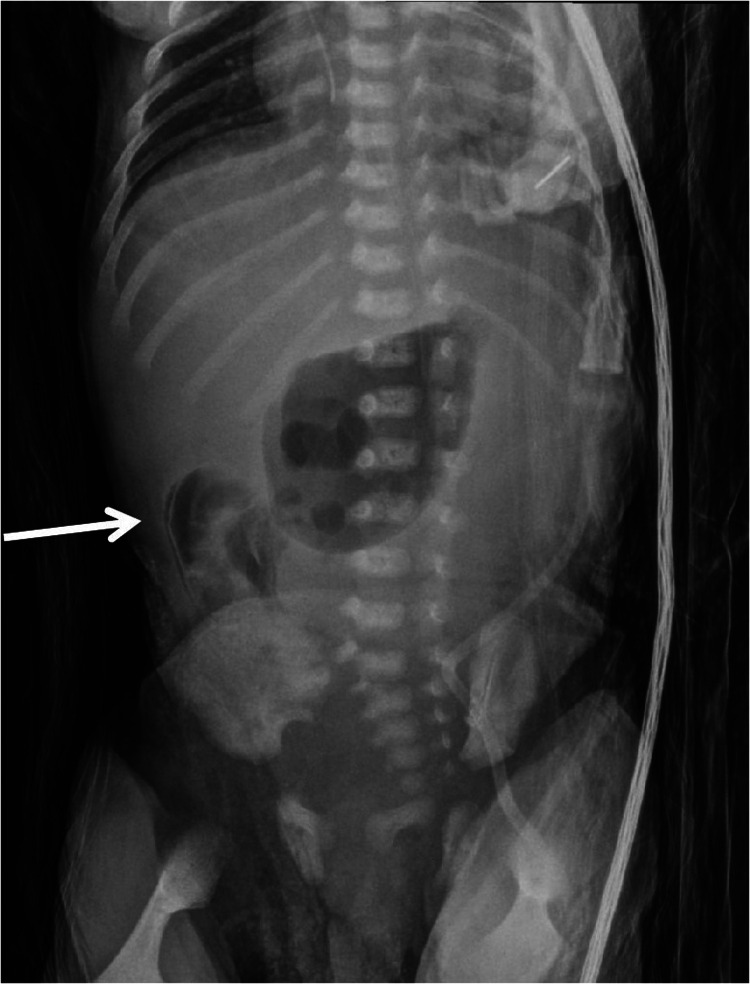
Plain radiograph shows multiple linear low-density gas shadows in the intestinal wall (as indicated by the arrows), consistent with the positions of the right descending colon and sigmoid colon (intraoperative confirmation).

Two weeks later, the newborn developed hematochezia, and a physical examination revealed mild abdominal distension, with no muscle rigidity and reduced bowel sounds. x-ray imaging demonstrated multiple linear, low-density gas shadows in the intestinal wall and liver area (Figure 3), leading to the diagnosis of NEC.Since no gastrointestinal perforation occurred and the infant's vital signs were stable, conservative treatment was chosen, including NPO and antimicrobial therapy.

After 4 days of conservative medical management, the newborn developed abdominal distension, signs of peritoneal irritation, and exhibited crying upon palpation. Reevaluation of infection markers revealed a progressive increase in C-reactive protein (CRP) and interleukin-6 (IL-6), along with a gradual decrease in platelet count (PLT). Plain radiographs continued to show gas in the intestinal wall, indicating that the inflammatory response had not been controlled and that conservative medical management had been ineffective. Consequently, surgical intervention was deemed necessary.A large amount of yellow, turbid fluid was found in the abdominal cavity. After thorough irrigation, extensive intestinal adhesions were identified. These adhesions were released, revealing the affected segment of the intestine. The descending colon was fixed on the posterior abdominal wall, shifted to the right from the splenic flexure to the midline. The entire colon is dilated with thickened walls. Extensive mucosal ulceration is present, accompanied by transmural necrosis. Postoperative pathology reveals significant infiltration of inflammatory cells.The most severely affected regions were the descending and sigmoid colon. A total colectomy with end ileostomy was performed, and the distal part of the sigmoid colon was preserved.

Five months after the surgery, the infant returned for stoma reversal. Preoperative gastrointestinal imaging revealed that the distal part of the sigmoid colon was blind, and the lumen was narrowed. Stoma reversal with anastomosis was successfully performed following resection of the sigmoid colon scar. The infant was discharged 18 days later. During the two-month follow-up period, no adverse complications were reported.

## 3 Discussion

NEC is a severe intestinal disorder that primarily affects premature infants, low birth weight (LBW) neonates, and those receiving enteral feeding. Despite extensive research, the pathogenesis of NEC remains incompletely understood. It is widely regarded as a multifactorial disease, with major contributing factors including intestinal immaturity, microbial dysbiosis, infection, genetic susceptibility, and impaired mucosal immune defense (3, 4). Recently, increasing attention has been given to the role of host–microbiota interactions in the development of NEC. According to this emerging concept, the absence of beneficial commensal bacteria, reduced microbial diversity, and overgrowth of pathogenic organisms contribute to microbial imbalance. This dysbiosis leads to excessive activation of Toll-like receptor 4 (TLR-4), resulting in an exaggerated inflammatory response, disruption of intestinal barrier function, localized ischemia, and ultimately, tissue necrosis (5–7).

In addition to microbial and immunological factors, anatomical variations of the colon may also influence intestinal function and disease susceptibility.SB Nayak first reported the discovery of displaced sigmoid and descending colons in the cadaveric body of an adult male approximately 65 years of age in 2013. In this case, the descending colon gradually shifted to the right from the splenic flexure to the midline, continuing to the sigmoid colon at the level of the fifth lumbar vertebra. The upper portion of the colon was attached to the upper part of the mesentery of the small intestine, while the lower part was anchored to the retroperitoneum. This anatomical variant is referred to as right-sided descending colon (8).

Under normal circumstances, by the 10th week of embryonic development, the midgut loop retracts into the abdominal cavity, with the jejunum formed by the head branch of the midgut first entering the first to enter the abdominal cavity. The preallantoic portion of the hindgut, originally located in the midline of the abdominal cavity, is pushed to the left side and develops into the descending colon and sigmoid colon. Therefore, the normal descending and sigmoid colons should be positioned on the left side of the abdomen (9).

However, when the jejunal loop fails to vacate the upper left quadrant during the formation of the splenic flexure, it may not move to the ventral and inferior sides as expected. This can result in mechanical obstruction, hindering the fusion of the primitive parietal peritoneum with the left wall of the mesocolon (10). At the same time,some scholars suggest that this abnormality arises due to secondary rotation of the large intestine, with the primary rotation occurring in the small intestine (11).

The occurrence of a right-sided descending colon is extremely rare, with only seven reported cases in the literature (8–14). Table 1 summarizes similar case reports.In all of these cases, the right descending colon was discovered incidentally, and no associated diseases were mentioned. Therefore, we believe that the right-sided descending colon itself does not typically present with clinical symptoms. Some scholars speculate that when the descending colon is filled with feces, it may compress the aorta, potentially causing changes in blood supply to the lower extremities (8). However, we did not find any relevant reports supporting this theory, and this patient did not exhibit such symptoms.

**Table 1:** Literature review of reported cases of right-sided colon.

Type	Age	Sex	Method of discovery	Lesion of right-sided descending colon	Lesion of sigmoid colon	Combined deformity	Country
Nayak SB 2013 (8)	65Y	M	Dissection of cadaver	N	N	N	India
Shrivastava P 2013 (10)	54Y	M	Dissection of cadaver	N	N	N	India
Cherian SB 2014 (11)	70Y	F	Dissection of cadaver	Descending colon exhibited segmental narrowing of lumen or stric ture along its length	N	The lengths of ascending and transverse colon were shorter than the normal.	India
Flores-Ríos E 2015 (13)	22Y	F	CT	N	N	Wandering spleen, gastric and pancreatic volvulus	Spain
Gül E 2017 (14)	54Y	M	Contrast-enhanced CT	N	Sigmoid Diverticulitis	N	Turkey
Gnansekaran D 2021 (9)	-	M	dissection of cadaver	N	N	N	India
Liang-Jing Lyu 2022 (12)	56Y	M	CT	N	Carcinoma located in sigmoid colon	N	China
Our case	2W	M	lower gastrointestinal imaging	necrotizing enterocolitis	necrotizing enterocolitis	N	China

Given that the right sigmoid and descending colon are generally asymptomatic and no related diseases have been reported, we do not consider surgical intervention necessary. However, it is important to note that the abnormal positioning of the colon may lead to clinical challenges in diagnosis and intervention. This variation can be diagnosed by CT and lower gastrointestinal imaging. If ectopic colon is found, the treatment method and surgical path should be adjusted according to the specific situation. For example, diverticulitis of the right-sided sigmoid colon may clinically resemble acute appendicitis, and awareness of this anatomical variation can help prevent unnecessary appendectomy (9). In addition, the right-sided descending colon is located directly in front of the right kidney. For surgeries involving areas covered by the ectopic descending colon, it is valuable for surgeons to be aware of this anatomical variation both pre- or intra-operatively. Furthermore, when performing colonoscopy or colon surgery, special attention must be given to the shape and positioning of the bowel to avoid unnecessary injury.

## 4 Conclusion

This case may help raise awareness among surgeons regarding the variability in the position of the descending colon. It is essential to consider such anatomical variations before performing related procedures in this area, such as colonoscopy and anterior transperitoneal aproach of kidney, to enhance surgical safety and avoid colonic injury.

## Data Availability

The raw data supporting the conclusions of this article will be made available by the authors, without undue reservation.

## References

[1] Fiorella DJ Donnelly LF. Frequency of right lower quadrant position of the sigmoid colon in infants and young children. Radiology. (2001) 219(1):91–4. 10.1148/radiology.219.1.r01ap3991 11274541

[2] Saxena AK Sodhi KS Tirumani S Mumtaz HA Narasimha Rao KL Khandelwal N. Position of a sigmoid colon in right iliac fossa in children: a retrospective study. J Indian Assoc Pediatr Surg. (2011) 16(3):93–6. 10.4103/0971-9261.83485 21897567 PMC3160061

[3] Samuels N van de Graaf RA de Jonge RCJ Reiss IKM Vermeulen MJ. Risk factors for necrotizing enterocolitis in neonates: a systematic review of prognostic studies. BMC Pediatr. (2017) 17(1):105. 10.1186/s12887-017-0847-3 28410573 PMC5391569

[4] Liu K Guo J Zhu Y Yang J Su Y. Analysis of risk factors and establishment of predictive models for neonatal necrotizing enterocolitis: a retrospective study. Ital J Pediatr. (2025) 51(1):80. 10.1186/s13052-025-01930-y 40087744 PMC11909941

[5] Cassir N Simeoni U La Scola B. Gut microbiota and the pathogenesis of necrotizing enterocolitis in preterm neonates. Future Microbiol. (2016) 11(2):273–92. 10.2217/fmb.15.136 26855351

[6] Cassir N Benamar S Khalil JB Croce O Saint-Faust M Jacquot A Clostridium butyricum strains and dysbiosis linked to necrotizing enterocolitis in preterm neonates. Clin Infect Dis. (2015) 61(7):1107–15. 10.1093/cid/civ468 26084844

[7] Thänert R Keen EC Dantas G Warner BB Tarr PI. Necrotizing enterocolitis and the microbiome: current status and future directions. J Infect Dis. (2021) 223(12 Suppl 2):S257–63. 10.1093/infdis/jiaa604 33330904 PMC8206796

[8] Nayak SB Pamidi N Shetty SD Sirasanagandla SR Ravindra SS Guru A Displaced sigmoid and descending colons: a case report. OA Case Reports. (2013) 2(17):166. https://www.oapublishinglondon.com/article/1072

[9] Gnansekaran D Prashant SA Veeramani R Yekappa SH. Congenital positional anomaly of descending colon and sigmoid colon: its embryological basis and clinical implications. Med J Armed Forces India. (2021) 77(2):241–4. 10.1016/j.mjafi.2019.10.006 33867645 PMC8042502

[10] Shrivastava P Tuli A Kaur S Raheja S. Right sided descending and sigmoid colon: its embryological basis and clinical implications. Anat Cell Biol. (2013) 46(4):299–302. 10.5115/acb.2013.46.4.299 24386604 PMC3875849

[11] Cherian SB Gandhalam AJ. Abnormal position of descending colon with right-sided sigmoid colon and its clinical significance. Apollo Medicine. (2014) 11(3):227–9. 10.1016/j.apme.2014.07.012

[12] Lyu LJ Yao WW. Carcinoma located in a right-sided sigmoid colon: a case report. World J Clin Cases. (2022) 10(18):6136–40. 10.12998/wjcc.v10.i18.6136 35949839 PMC9254206

[13] Flores-Ríos E Méndez-Díaz C Rodríguez-García E Pérez-Ramos T. Wandering spleen, gastric and pancreatic volvulus and right-sided descending and sigmoid colon. J Radiol Case Rep. (2015) 9(10):18–25. 10.3941/jrcr.v9i10.2475 PMC4638397 26629290

[14] Gül E Gül Y Gönen AN Özkan Z Bozdağ PG Aslan Y. Sigmoid diverticulitis mimicking acute appendicitis in right-sided descending and sigmoid colon: a case report. J Emergency Med Case Reports. (2017) 8(1):16–9. 10.5152/jemcr.2016.1538

